# Efficient Likelihood-Based Temporal Changepoint Detection in Spatio-Temporal Processes

**DOI:** 10.1007/s11222-025-10745-0

**Published:** 2025-10-17

**Authors:** Gaurav Agarwal, Idris A. Eckley, Paul Fearnhead

**Affiliations:** Department of Mathematics and Statistics, Lancaster University, Bailrigg, Lancaster, LA1 4YW United Kingdom

**Keywords:** Changepoint, Markov approximation, Nonstationarity, Spatiotemporal data, Wind speed

## Abstract

The rapid advancements of scalable methodologies have opened new avenues for analyzing complex spatio-temporal data, which is crucial in understanding dynamic environmental phenomena. This paper introduces a likelihood-based methodology for detecting abrupt changes in time in spatio-temporal processes, a field where traditional time series methods fall short. Unlike recent approaches, we do not make the unrealistic assumption that data is independent across changepoints. Instead, we use a recently proposed family of covariance models that allows nonstationarity in time, and we propose a Markov approximation to reduce the computational burden of calculating likelihoods under this model. We apply our method to two years of daily wind speed data from various synoptic weather stations in Ireland, identifying a significant changepoint on July 24, 2021, which aligns with a major shift in weather patterns. This application not only demonstrates the method’s utility in handling spatio-temporal datasets but also showcases its potential in broader environmental and climatic studies, offering a scalable solution for analyzing changing patterns in spatial data over time.

## Introduction

Recent developments in statistical methodologies have significantly enhanced our ability to analyze complex spatio-temporal data, particularly in environmental science. However, traditional methods often assume stationarity when modelling spatio-temporal data, an assumption that is increasingly recognized as inappropriate in many real-world scenarios (Stroud et al. 2001; Fuentes et al. 2008; Sigrist et al. 2012; Ezzat et al. 2019). This manuscript introduces a likelihood-based methodology for detecting temporal changepoints in spatio-temporal processes. Our proposed framework focuses on identifying changepoints in time at which the entire spatial covariance (or mean) structure shifts. Our approach innovatively adapts a nonstationary model over time, incorporating a Markov approximation to address the computational challenges commonly associated with full likelihood models. Our work is distinct in its focus on changepoint analysis for spatio-temporal data, a domain that has remained relatively unexplored compared to its univariate and multivariate counterparts.

Changepoint analysis has evolved significantly over the years, with extensive research conducted on univariate time series data (Fearnhead and Liu 2007; Chen and Gupta 2012; Killick and Eckley 2014; Haynes et al. 2017; Hocking et al. 2022). The methodology has also expanded into multivariate and high-dimensional data arenas (Matteson and James 2014; Arlot et al. 2019; Cho and Fryzlewicz 2015; Wang and Samworth 2018; Enikeeva and Harchaoui 2019; Tickle et al. 2021), as well as into functional data (Aston and Kirch 2012; Aue et al. 2018). Each of these approaches, while innovative, often relies on assumptions that may not hold in spatio-temporal contexts. For instance, traditional models typically assume independence between data in segments separated by changepoints, or apply simplistic covariance structures that fail to capture the intricacies of spatio-temporal dependencies.

We consider the setting where we have data at a set of discrete time-points, with, at each time-point, measurements associated with a set of spatial locations. The number of measurements and their location can vary between time-points. We want to allow for dependencies in data across time and space, and to detect time-points where there is an abrupt change in either the mean or covariance structure (or both) of the data. There has been work on modelling nonstationarity in spatio-temporal data (for example Garg et al. 2012; Shand and Li 2017; Salvaña and Genton 2021) but such approaches are not specifically tailored to detecting abrupt changes or estimating the time that changes occur.

There is a growing body of methods for changepoint detection in spatio-temporal methods. However, common limitations include the assumption of separability in spatial and temporal covariance functions (Majumdar et al. 2005; Altieri et al. 2015; Gromenko et al. 2017). This assumption simplifies the computational process but at the cost of ignoring real-world data complexities where space-time interactions are crucial. The method of Zhao et al. (2019) uses nonseparable covariance functions but then assumes that data in segments separated by changepoints are independent. This assumption is unrealistic and can lead to inconsistencies and errors in changepoint detection, particularly as real environmental data exhibit intricate temporal dependencies.

Motivated by the limitations of these existing methods, we present an approach that models the data using non separable covariance functions but that also preserves temporal dependence across change-points. This is achieved by using recently proposed non separable covariances that are able to evolve over time (Qadir and Sun 2023). We then model the time-varying parameters of this covariance function as piece-wise constant functions over time. Similarly, we introduce a model for the mean that can vary abruptly over time. Points where either the covariance parameters, the mean, or both, change are changepoints. We first consider a single changepoint model, and derive a likelihood ratio test for whether there is change and, if there is, the time at which it occurs. This procedure can then be embedded within binary segmentation so as to detect multiple changepoints.

One challenge with our approach is that the cost of calculating the likelihood scales cubically with the number of data-points, i.e. the number of pairs of time and location for which we have measurements. This can be computationally prohibitive. To overcome these computational issues, we implement a Markov approximation. As suggested by Cressie and Wikle (2015), most real-world spatio-temporal processes can be characterized conditional on the process in the recent past. This suggests approximating the probability density of data at time *t* given all earlier data by its conditional density given data at the *k* most recent time-points. This reduces the computational cost of calculating the likelihood from being cubic in the number of time-points to cubic in *k*. Recent studies like Alyousifi et al. (2020) demonstrate the efficacy of such an approximation for environmental time-series. In the case of large time series, we also propose using the optimistic search strategy (Kovács et al. 2020) to further speed up the changepoint search. This method adaptively determines which times to test for a change, instead of searching through all possibilities.

**Fig. 1:**
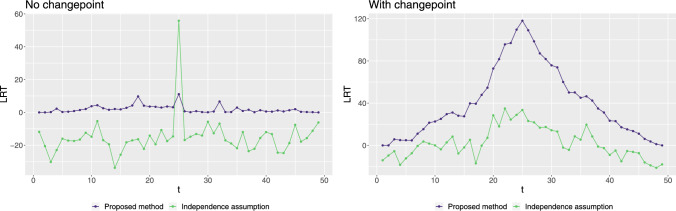
Likelihood ratio test statistics to detect a single changepoint in the spatial process using the proposed method and independent segment method for two simulation settings of spatio-temporal data (i) no changepoint but independent segments at $t=25$, (ii) changepoint at $t=25$

To illustrate the issues of assuming independence across segments when detecting changepoints in a spatio-temporal context that our approach overcomes, Figure 1 compares two approaches on two simulated datasets of length 50. At each candidate time index $\tau$, we compute a likelihood ratio statistic (LRS) for testing:

$$ H_0: \text {no change at } \tau \quad \text {vs.} \quad H_1: \text {change at } \tau . $$

Under our proposed method, we use the nonstationary spatio-temporal framework from Section 2.2, which maintains a valid joint covariance across time and does not force the process to be independent before and after $\tau$. In contrast, mimicking the independent segment approach of Zhao et al. (2019) artificially treats the data before time $\tau$ and after time $\tau$ as independent.

In the first simulation of Figure 1, we merged realizations of two independent spatio-temporal processes with the same mean and covariance. Thus there is no change, but there is independence between data for $t\le 25$ and for $t>25$. In the second simulation, we introduce an actual shift in the spatial range at $t=25$ using our proposed nonstationary model.

We observed two issues with the approach that assumes independence of data in different segments. The first is that the change point model is not nested within the no-change model, so the likelihood-ratio statistic can be negative. Consequently, standard properties of the likelihood-ratio test may not hold. More importantly, we see that the main signal from the test comes from the independence across the change – with a clear spike in the test statistic at $t=25$ when the data before and after $t=25$ simulated independently, even though from the same model. Conversely much less evidence for a change is observed when the spatio-temporal model itself changes. By comparison, our approach, which does not make this independence assumption is able to correctly detect only the change in the spatio-temporal process.

The paper is organized as follows. Section 2.1 introduces background and notation, which lays the foundation for understanding the advanced techniques used in our approach. Section 2.2 details the proposed changepoint model approach for spatio-temporal processes, explaining how it adapts to the unique challenges of nonstationarity and dependency in data. In Section 2.3, we delve into the specifics of the Markov likelihood approximation, discussing its implementation and benefits. Section 2.4 expands on this by discussing the extension of our methodology to detect multiple changepoints, which is crucial for handling complex datasets. Comprehensive simulation studies are presented in Section 3, which validate the effectiveness of our approach against both synthetic and mimicking real-world scenarios. We then move to Section 4, where we apply our methodology to a case study involving wind data from Ireland, illustrating the practical implications of our findings. Finally, Section 5 provides concluding remarks, highlighting the contributions of our work to the field and suggesting directions for future research.

## Methodology

### Background and notation

Let $\{Y(\varvec{s},t): \varvec{s} \in {\mathbb {R}}^d, t \in \{t_1, \ldots , t_T\} \}$ denote a spatio-temporal process where $\varvec{s}$ is a location of interest in ${\mathcal {S}} \subset {\mathbb {R}}^d$, and *t* is a discrete time. To ease presentation, in the following we will assume $t\in \{1,2,\ldots ,T\}$, but the ideas extend to non-regularly sampled data. To develop ideas, we will also assume that for each time point there are *m* spatial observations as locations $\varvec{s}_1,\ldots ,\varvec{s}_m$. Our method extends simply to situations where we observe different numbers of observations, at possible different locations, at each time-point. Then the random vector of all observations is given by $\varvec{Y} = \big ( Y(\varvec{s}_1,t_1),\dots , Y(\varvec{s}_m,t_1), \dots , Y(\varvec{s}_1,t_T), \dots , Y(\varvec{s}_m,t_T) \big )^\top$, with $n=mT$ total observations. We assume that the spatio-temporal process follows a Gaussian process model. This is a typical choice in spatio-temporal modelling where the joint distribution of variables indexed in space and time is multivariate normal. The vector $\varvec{Y} \sim \mathcal {MVN} (\varvec{\mu }_{n\times 1}, \varvec{\Sigma }_{n\times n})$, where $\varvec{\mu } = [ E\{Y(\varvec{s}_1,t_1)\}, \dots , E\{Y(\varvec{s}_m,t_T)\}]$ and $\varvec{\Sigma } = [ \text {Cov}\{Y(\varvec{s}_i,t_i), Y(\varvec{s}_j,t_j) \} ]_{i,j=1}^n$ are the usual mean vector and covariance matrix of the multivariate normal distribution, respectively. The entries of the covariance matrix $\varvec{\Sigma }$ are usually defined through a non-negative definite parametric covariance function. Among the existing nonseparable spatio-temporal covariance functions, Gneiting (2002)’s class of covariance functions are the most widely used and allow for space-time interaction. Their covariance structure is assumed to be stationary in space and time, so the covariance is dependent only on the space-time lag, denoted by $(\varvec{h}, u)$ respectively. Gneiting (2002)’s class of stationary space-time covariance functions is given by

1 $$\begin{aligned} C(\varvec{h}, u) = \frac{\sigma ^2}{ \psi (|u|^2)^{d/2}} \phi \Bigg ( \frac{\Vert h\Vert ^2}{ \psi (|u|^2)}\Bigg ), \quad (\varvec{h}, u) \in {\mathbb {R}}^d \times {\mathbb {R}}, \end{aligned}$$

where $\sigma$ is the marginal standard deviation of the process, $\phi (w), w\ge 0$ is any completely monotone function, and $\psi (w), w \ge 0$, is any positive function with a completely monotone derivative, commonly termed as a Bernstein function. Common choices of $\phi (\cdot )$ and $\psi (\cdot )$ are given in tables 1 and 2 of Gneiting (2002). Though the covariance function is nonseparable, $\phi (\cdot )$ can be associated with the data’s spatial structure and $\psi (\cdot )$ with temporal structure. For concreteness, $\phi (w) = (c w^{1/2})^\nu K_{\nu }(c w^{1/2})/\{ 2^{\nu -1} \}$, $c>0$, $\nu >0$, where $K_\nu (\cdot )$ denotes a modified Bessel function of the second kind of order $\nu$ and $\psi (w) = (aw^\alpha +1)^\beta , a>0, 0< \alpha \le 1, 0 \le \beta \le 1$ are the particular choices we consider in this paper. For these choices, a purely spatial covariance function in (1) is given by $C(\varvec{h}, 0) = \sigma ^2 (c \Vert h\Vert )^\nu K_\nu (c \Vert h\Vert ) 2^{1-\nu }/ \Gamma (\nu )$. This is a Matérn covariance function, denoted as $\sigma ^2M(\varvec{h} \mid c, \nu )$, where $c> 0$ and $\nu >0$ are spatial scale and smoothness parameters, respectively. To define a changepoint model, we cannot use Gneiting (2002)’s covariance function because it is not flexible enough to allow for a change unless the independent segment assumption is made. However, Qadir and Sun (2023) defined a generalization of Gneiting’s stationary space-time covariance function which allows for time-evolving spatial parameters. It is this generalization that we will use in the next section to define valid changepoint models.

### Spatio temporal changepoint model

Consider a spatio-temporal process

2 $$\begin{aligned} Y(\varvec{s}, t) = \mu (\varvec{s}, t) + \epsilon (\varvec{s}, t), \end{aligned}$$

$t = 1,\dots , T$ and $\varvec{s} \in {\mathcal {S}}$, where $\mu (\varvec{s}, t)$ is the deterministic mean and $\epsilon (\varvec{s}, t)$ is the mean-zero error process. The mean can be a constant, $\mu$, or a regression of a form such as $z_{t, \varvec{s}}^\top \beta$, where $z_{t, \varvec{s}}$ are covariates associated with $(\varvec{s}, t)$. The covariance structure is given by $\text {Cov}\{ Y(\varvec{s},t_i), Y(\varvec{s}+ \varvec{h},t_j)\} = C(\varvec{h}, t_i, t_j)$, a non separable, nonstationary in time covariance function, which allows the spatial process to evolve over time (Qadir and Sun 2023). Following Qadir and Sun (2023), the generalized parametric form of the covariance function is given by

3 $$\begin{aligned} C(\varvec{h}, t_i, t_j)&= \sigma ^2 \frac{\Gamma \{\frac{\nu _{s}(t_i)+\nu _s(t_j)}{2}\}}{\sqrt{\Gamma \{\nu _s(t_i)\}\Gamma \{\nu _s(t_j)\}}} \frac{f_{\psi , c_s}(t_i,t_j)}{c_s(t_i)c_s(t_j) }\nonumber \\&M\left[ \varvec{h} \mid f^{1/2}_{\psi , c_s}(t_i,t_j), \frac{\nu _s(t_i)+\nu _s(t_j)}{2}\right] , \end{aligned}$$

where $$f_{\psi , c_s}(t_i,t_j) = 1/ \{ \frac{\psi (|t_i-t_j|^2)}{\bar{c}_{\varvec{s}}^2} + \frac{1/c^2_{\varvec{s}}(t_i) +1/c^2_{\varvec{s}}(t_j)}{2}- \frac{\psi (0)}{\bar{c}_{\varvec{s}}^2}$$ }, with $$\bar{c}_{\varvec{s}} = \sum _{t_i} c_s(t_i)/T $$ and is valid for any $$c_s(t) >0 $$, $$\nu _s(t) >0 $$ positive real valued functions and any Bernstein function $$\psi (w) >0$$, $$w \ge 0$$. Note that $$ C(\varvec{h}, t_i, t_j)$$ in (3) generalizes many popular covariance functions. For example, if $$c_s(t) = c>0, \nu _s(t) = \nu > 0$$, then $$\bar{c}_{\varvec{s}} = c$$ and (3) reduces to Gneiting (2002)’s space time covariance function. Alternatively, for $$t_i=t_j$$, (3) becomes the Matérn covariance function at time *t*;

4 $$\begin{aligned} C_t(\varvec{h})= &  \frac{\sigma ^2}{2^{\nu _s(t)-1}} \big (c_s(t) \varvec{h}\big )^{\nu _s(t)} K_{\nu _s(t)}\big (c_s(t) \varvec{h}\big )\nonumber \\= &  \sigma ^2 M(\varvec{h} \mid c_s(t), \nu _s(t)), \end{aligned}$$

the Matérn class where $c_s(t)$ and $\nu _s(t)$ are time-varying spatial scale and smoothness parameters. Further, if we set $\nu _s(t) = 0.5$ in (4), it reduces to an exponential covariance function. Also note that if $\nu _s(t) = \nu \rightarrow \infty$, then the Gaussian covariance function is obtained.

To get some intuition for (3), consider fixing $t_i\ne t_j$ and viewing the covariance as a function of $\varvec{h}$. This spatial covariance between time $t_i$ and $t_j$ is Matérn but with a scale $f_{\psi , c_s}$ and smoothness $[\nu _s(t_i)+\nu _s(t_j)]/2$ which depend on $t_i$ and $t_j$. The terms outside the $M[\cdot |\cdot ,\cdot ]$ function then specify the overall level of covariance between these two times. In our use of this covariance function we will model $\nu _s(\cdot )$ and $c_s(\cdot )$ to be piecewise constant over time. In the absence of a changepoint, these are constant and, as mentioned above, the covariance is given by Gneiting (2002)’s nonseparable covariance function.

Having established the approach to model the covariance structure, we now consider the problem of detecting a changepoint in a spatio-temporal process. Our objective is to detect a change in the spatial process over time. The spatial process at time *t* is parameterised by mean parameters, denoted by $\varvec{\mu }_t$ and covariance parameters spatial scale and smoothness, denoted by $\varvec{\theta }_t$. We are interested in testing whether there is a single changepoint in the mean or covariance of the spatial process at $\tau \in \{1,2,\dots , T-1\}$. To this end, we define the null hypothesis of no change in the spatial process as

$$\begin{aligned} H_0: \varvec{\mu }_1 = \varvec{\mu }_2 = \dots \varvec{\mu }_T \quad \text {and}\quad \varvec{\theta }_1 = \varvec{\theta }_2 = \dots \varvec{\theta }_T = \varvec{\theta }_0, \end{aligned}$$

against the alternative hypothesis with a change in mean or spatial covariance

$$\begin{aligned} H_{A}: \varvec{\mu }_1 = \dots \dots \varvec{\mu }_{\tau } = \varvec{\mu }^{(1)} \ne \varvec{\mu }_{\tau +1} = \dots \varvec{\mu }_T = \varvec{\mu }^{(2)}, \quad \text {or } \quad \\ \varvec{\theta }_1 = \dots \dots \varvec{\theta }_{\tau } = \varvec{\theta }^{(1)} \ne \varvec{\theta }_{\tau +1} = \dots \varvec{\theta }_T = \varvec{\theta }^{(2)}. \end{aligned}$$

Under the alternative hypothesis, the change in mean is denoted as $\mu (\varvec{s}, t_i)$ equals $\mu _1$ if $t_i \le \tau$ and equals $\mu _2$ if $t_i > \tau$. For the spatial covariance parameters, a change would be denoted as

$$\begin{aligned} c_s(t_i) = {\left\{ \begin{array}{ll} c_1,~~ t_i \le \tau \\ c_2, ~~t_i> \tau \end{array}\right. } \quad \text {and} \quad \nu _s(t_i) = {\left\{ \begin{array}{ll} \nu _1, ~~ t_i \le \tau \\ \nu _2, ~~t_i> \tau . \end{array}\right. } \end{aligned}$$

Here $\tau$ is an unknown location of the changepoint, where $\mu _1$, $c_1$ and $\nu _1$ are mean and covariance parameters of the segment before the change and $\mu _2$, $c_2$ and $\nu _2$ after the change. Let us next consider a likelihood ratio test statistic for testing a changepoint in the spatial process. We are primarily interested in testing the changes in parameters $\varvec{\mu }_t$ and $\varvec{\theta }_t$, while treating $\gamma = \{ a, \alpha , \beta \, \sigma ^2\}$ as the nuisance parameters that encompass both temporal parameters and marginal variance. Under the alternative hypothesis, the likelihood ratio test statistic is given by

5 $$\begin{aligned} \text {LR}_\tau= &  -2 \log \nonumber \\  &  \Bigg [ \frac{\underset{ \varvec{\mu }, \varvec{\theta _0},\varvec{\gamma }_0 }{\text {max}} f_{\varvec{Y}}(\varvec{y}; \varvec{\mu }, \varvec{\theta }_0,\varvec{\gamma }_0 ) }{\underset{ \varvec{\mu }^{(1)},\varvec{\mu }^{(2)}, \varvec{\theta }^{(1)}, \varvec{\theta }^{(2)},\varvec{\gamma }_1 }{\text {max}} f_{\varvec{Y}}\{\varvec{y}; \varvec{\mu }^{(1)},\varvec{\mu }^{(2)}, \varvec{\theta }^{(1)}, \varvec{\theta }^{(2)}, \varvec{\gamma }_1 \} } \Bigg ],\nonumber \\ \end{aligned}$$

where $f_{\varvec{Y}}(\cdot )$ is the multivariate Gaussian density function. In practice, maximization is implemented through numerical optimization. Since the changepoint location is unknown, the likelihood ratio test statistic is evaluated at $\tau = 1,2, \dots , T-1$, and the maximum is considered, i.e., $\text {LR} = \underset{\tau \in \{1,\dots ,T-1\}}{\text {max}} \text {LR}_\tau$. We use the Monte Carlo method to estimate the distribution of test statistics under the null hypothesis, (which provides a good approximation of the distribution of ${\text {max}} \, \text {LR}_\tau$, see Hawkins 1977), and use this to choose an appropriate threshold for our test. The obtained threshold denoted *c*, also determines the significance level of the test. If $\text {LR} > c$, then a changepoint is detected and the estimate of the location of the changepoint is given by

6 $$\begin{aligned} \hat{\tau } = \text {arg} \underset{\tau \in \{1,\dots ,T-1\}}{\text {max}} \text {LR}_\tau . \end{aligned}$$

The above provides the general methodology for detecting a change in spatial mean or covariance, but the methodology can be easily adapted to test for a change only in the spatial mean or spatial dependence. For example, to test for a change in spatial mean only, the covariance parameters are fixed in the alternative hypothesis, while for a change in spatial covariance only, the mean parameters are fixed. The likelihood ratio statistic in (5) is adjusted accordingly and the rest of the procedure remains the same.

This section has introduced a new procedure for fitting nonstationary, spatio-temporal models that detects the presence of changepoints and can also be used for spatio-temporal prediction which is often the objective in many studies. The proposed changepoint model is flexible, allowing for changes in the spatial process, however, the estimation of parameters with the full likelihood is computationally challenging. The main issue lies in storing and inverting large spatio-temporal covariance matrix in the full likelihood, which makes the computation infeasible. To overcome this, we implement a Markov likelihood-based procedure in the next section.

### Markov likelihood and estimation

In this section, we introduce the Markov approximation to the likelihood procedure. To this end, the log-likelihood of $\varvec{Y}$ is given as: $\ell (\varvec{\mu },\varvec{\theta } \mid \varvec{Y}) = -\{ \text {log} \, \text {det} \Sigma (\varvec{\theta }) + (\varvec{Y} - \varvec{\mu })^\top \Sigma (\varvec{\theta })^{-1} (\varvec{Y} - \varvec{\mu }) + n\text {log} 2\pi \}/2$, where $\Sigma (\varvec{\theta })$ is the $n \times n = mT \times mT$ covariance matrix for $\varvec{Y}$, defined through a spatio-temporal covariance function with parameters $\varvec{\theta }$. The parameters are estimated using the maximum likelihood method, which involves numerical optimization. Although full likelihood achieves high statistical efficiency, it involves the inverse of a high-dimensional covariance matrix. The optimization becomes increasingly challenging in case both or either of *m* and *T* are large as the dimensions of the covariance matrix $\Sigma (\varvec{\theta })$ become large. Storing a very large matrix can also exhaust the memory of the machine, making it computationally impractical.

To deal with the computational issues, we implement a Markov approximation of the likelihood, which provides a substantial simplification of the full likelihood, and yet is able to model a process that is complicated (Cressie and Wikle 2015). Let $\varvec{Y}_t(\cdot )$ denote the spatial process at time *t*. The log-likelihood of $\varvec{Y}$ can be represented as $\ell (\cdot \mid \varvec{Y}) = \ell \{\cdot \mid \varvec{Y}_1(\cdot ), \dots ,\varvec{Y}_T(\cdot ) \} = f_{\varvec{Y}_1(\cdot ), \dots ,\varvec{Y}_T(\cdot )}$, the joint distribution of $\varvec{Y} = \{ \varvec{Y}_1(\cdot ), \dots ,\varvec{Y}_T(\cdot ) \}^\top$. We decompose the joint distribution of the process in terms of conditional distributions that respect the time evolution of the spatial process. In particular, the joint distribution of $\varvec{Y}_1,\dots ,\varvec{Y}_T$ can be represented using the chain rule of conditional probabilities

7 $$\begin{aligned} \{ \varvec{Y}_1,\dots ,\varvec{Y}_T \}&= \{\varvec{Y}_{T} \mid \varvec{Y}_{T-1}, \dots ,\varvec{Y}_1\} \dots \{\varvec{Y}_{3} \mid \varvec{Y}_{2},\varvec{Y}_1\} \nonumber \\ &\quad \{\varvec{Y}_{2} \mid \varvec{Y}_{1}\} \{\varvec{Y}_{1}\}. \end{aligned}$$

The joint distribution in (7) can be simplified using a Markov assumption, in which the conditional probabilities of the process at time *t*, conditioned on the process at times prior to *t*, is only dependent on the most recent time. For example, a first-order Markov assumption is given by

$$\begin{aligned} \{\varvec{Y}_{t} \mid \varvec{Y}_{t-1}, \dots ,\varvec{Y}_1\} = \{\varvec{Y}_{t} \mid \varvec{Y}_{t-1} \}, \quad t=2,\dots , T. \end{aligned}$$

Consequently, adopting the first-order Markov assumption, the joint distribution of equation (7) simplifies to

8 $$\begin{aligned} \{\varvec{Y}_1,\dots ,\varvec{Y}_T\} = \prod _{t=2}^T \{\varvec{Y}_t\mid \varvec{Y}_{t-1}\} \times \{\varvec{Y}_{1}\}. \end{aligned}$$

Notice from (8) that the complicated joint distribution can be modeled by relatively simple conditional distributions, provided this assumption is valid. In this case, we have to invert an $m \times m$ matrix *T* times. Hence, the computational complexity of Markov likelihood is reduced to $O(m^3T)$ from full likelihood complexity of $O(m^3T^3)$.

We can extend this idea to a *k*th order Markov assumption by approximating, for $t>k$ the distribution $\{\varvec{Y}_{t} \mid \varvec{Y}_{t-1}, \dots ,\varvec{Y}_1\}$ by $\{\varvec{Y}_{t} \mid \varvec{Y}_{t-1}, \dots ,\varvec{Y}_{t-k}\}$. For such a $k^{\text {th}}$ order Markov assumption, the complexity scales $k^3$ times of the first-order Markov likelihood complexity. Thus, adopting the Markov likelihood approach, the computation and optimization of $\ell (\varvec{\mu },\varvec{\theta } \mid \varvec{Y})$ is more feasible as it includes smaller sized covariance matrices of dimension $km \times km$. For example, in the Ireland wind data application, we have $m=22$ and $T= 731$. Hence, in this example, adopting a full likelihood involves inverting a matrix of size $16082 \times 16082$, while a third-order Markov likelihood involves inverting matrices of size $66 \times 66$.

The robustness of the Markov likelihood approach in handling missing data is a key advantage, particularly for Gaussian processes where the method relies on conditional Gaussian distributions. This flexibility is advantageous as it allows for the construction of conditional distributions using available observations, mitigating the impact of missing data points. However, the extent of missing data, particularly at recent time steps, could potentially affect the accuracy of the approximations. If a significant number of spatial locations are missing at time $t-1$, it may be necessary to consider information from an earlier time step, such as $t-2$, to achieve a more accurate conditional distribution for time *t*. While the Markov likelihood approach efficiently manages large spatio-temporal datasets, it encounters limitations when dealing with non-linear dynamics or long-range dependencies. The presence of noise in the observations may influence the decision on the number of lags to include in the model. A higher noise level might necessitate a greater number of lags to capture the underlying process accurately. These considerations underscore the need for careful model specification and the potential for choosing the order of the Markov assumption based on the patterns of data at hand.

### Multiple changepoints

We combine our proposed model of estimating a single changepoint in spatio-temporal processes with binary segmentation to detect multiple changepoints sequentially. Binary segmentation is a well-established approach to extend single change point methodologies to detect multiple changepoints (Scott and Knott 1974). The main idea is to first search for a single changepoint in the entire time series using, for instance, a likelihood-based method. If a changepoint is detected, the data is split into two segments, divided at the changepoint. A similar search is performed on both resulting segments, possibly resulting in further splits. The procedure is repeated until no further changepoints are detected. For the spatio-temporal setting, spatial data is observed at different time points represented by $\varvec{Y}_1,\dots , \varvec{Y}_T.$ The procedure to detect multiple changepoints with binary segmentation is explained in Algorithm 1.

As in the single-changepoint case, $LR(\varvec{Y}_{s:e})$ searches over all possible time points within [*s*, *e*], returning the maximum likelihood ratio value; likewise, $\hat{\tau }(\varvec{Y}_{s:e})$ identifies the time point at which that maximum occurs. We stop partitioning a given segment [*s*, *e*] once its LR statistic is below the threshold *c*, indicating no significant change within that interval. Otherwise, we split [*s*, *e*] at $\hat{\tau }$ and repeat the same test procedure recursively on the new subintervals.
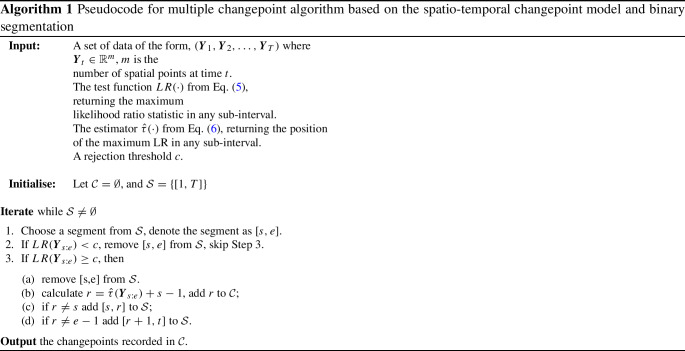


For a spatial process with large time series, searching through all the candidate changepoints to find the best one can be computationally demanding, as each point requires fitting a spatio-temporal model. In such a case, the optimistic search strategy can be used to speed up the computation (Kovács et al. 2020). Specifically, instead of evaluating every candidate point, the interval is divided into three subintervals, and one outer subinterval is discarded after comparing likelihood ratios. The key idea is to adaptively determine the next search point given the previous one by splitting the search interval into three segments recursively and discarding one of the outer segments in each iteration. This leads to $O(\text {log} T)$ evaluations as compared to *O*(*T*) for a full grid search, which is a massive computational gain. Further, this optimistic search strategy combined with binary segmentation, termed optimistic binary segmentation, can be used to detect multiple changepoints.

## Simulation study

In this section, we conduct a simulation study to empirically evaluate the performance of the proposed spatio-temporal changepoint model based on Markov-likelihood estimation. We study the effect of Markov approximation and how it compares with the full likelihood approach. We also compare the proposed method with the independent segment method considering the pairwise likelihood approach (Zhao et al. 2019). We consider both a true model and a misspecified model and compare the methods on changepoint detection performances.

**Table 1:** Evaluation of changepoint detection methods for a single changepoint in the spatial range. The table compares the performance, measured in terms of Power and True Positive Rate (TPR) across different sizes of change in the spatial range parameter. Standard errors from 100 simulations are shown in brackets

size of change	Method	Power	TPR
0.025	Proposed (Markov Lik)	0.70 (0.05)	0.40 (0.05)
	Proposed (Full Lik)	0.74 (0.04)	0.43 (0.05)
	Ind Segment (Pair Lik)	0.00 (0.00)	0.00 (0.00)
0.05	Proposed (Markov Lik)	1.00 (0.00)	0.91 (0.03)
	Proposed (Full Lik)	1.00 (0.00)	0.95 (0.02)
	Ind Segment (Pair Lik)	0.40 (0.05)	0.10 (0.03)
0.2	Proposed (Markov Lik)	1.00 (0.00)	1.00 (0.00)
	Proposed (Full Lik)	1.00 (0.00)	1.00 (0.00)
	Ind Segment (Pair Lik)	0.70 (0.05)	0.20 (0.04)
0.3	Proposed (Markov Lik)	1.00 (0.00)	1.00 (0.00)
	Proposed (Full Lik)	1.00 (0.00)	1.00 (0.00)
	Ind Segment (Pair Lik)	0.95 (0.02)	0.50 (0.05)

In the first instance, we simulate a zero-mean Gaussian spatio-temporal process with a time-evolving space-time covariance function (Qadir and Sun 2023) as described in equation (3). We consider an irregularly spaced grid over the region $[0, 1] \times [0, 1]$. At time *t*, we consider exponential covariance function given by: $C_t(\varvec{h}) = \sigma ^2 \text {exp} \{ -c_t||\varvec{h}||\}$, where $c_t$ is the spatial scale at *t*, and $\sigma ^2$ is the overall variance. We choose $\psi (w) = (aw^\alpha +1)^\beta$ in the spatio-temporal covariance function (3) which is associated with temporal dependence.

Specifically, we fix $a=0.5$, $\alpha =0.5$, $\beta =0.7$, and $\sigma =1$, and let

$$ c_t = {\left\{ \begin{array}{ll} c_1 & \text {if } t \le 25,\\ c_1 + \delta & \text {if } t > 25, \end{array}\right. } \quad \text {where } c_1=1. $$

Thus, before $t=25$, the spatial scale is $c_1=1$ (range $=1$), and after $t=25$, it becomes $1+\delta$ (range $=1/(1+\delta )$). Our goal is to detect this abrupt change at $t=25$.

Initially, let us consider a single changepoint scenario. We set the covariance parameters as $a = 0.5$, $\alpha =0.5$, $\beta = 0.7$, $\sigma = 1$, and simulate a Gaussian process with $m=25$ and $T=50$ with a single changepoint in spatial scale at $t=25$. Spatial range is denoted by one over the spatial scale, $1/c_t$. The spatial range is the distance beyond which the spatial dependence of the process is negligible. For the simulation setting over the region $[0, 1] \times [0, 1]$, the maximum spatial range can be $\sqrt{2} = 1.41$. We vary the size of changes in the spatial range using the specific values of 0.025, 0.05, 0.2, and 0.3, and repeat the simulations 100 times. Throughout the simulation studies, the threshold for the likelihood ratio test is computed using the Monte Carlo method at a significance level of 0.05. Specifically, we generate 100 Monte Carlo samples from the null model (no change in the spatial scale) to approximate the distribution of the test statistic under $H_0$.

To evaluate the performance of changepoint methods, we compute (i) the power of the test, the proportion of times a change is detected when there is a change and (ii) the true positive rate (TPR), i.e., the proportion of the correctly identified changepoints among the estimated changepoints. In addition to reporting the average values of power and TPR, we also provide their Monte Carlo standard errors (computed as $\sqrt{{\hat{p}}(1-{\hat{p}})/100}$), where $\hat{p}$ denotes the empirical proportion of successes (power or TPR). These standard errors quantify the uncertainty due to the finite number of simulation replications. These metrics used to evaluate the changepoint detection methods are crucial for understanding their effectiveness in practical scenarios. Power refers to the method’s ability to correctly identify the presence of a changepoint when one truly exists. High power indicates a high likelihood that the method will detect a changepoint when it is present, which is crucial for not missing significant changes in the data that could have practical implications. TPR, on the other hand, measures the accuracy with which the detected changepoints correspond to actual changes, indicating the precision of the method. High TPR is essential for ensuring that the detected changepoints are not false positives. The results for detecting a change in spatial range for the proposed method with first-order Markov likelihood, full likelihood and the independent segment method are shown in Table 1. It can be seen that the performance of the Markov likelihood is close to that of the full likelihood approach, and it outperforms the independent segment method. For this simulation setting, we note that the Markov-likelihood approach is roughly 12 times faster than the full-likelihood approach in these simulations. This speed-up factor was observed on a standard desktop (2.6 GHz CPU, 16 GB RAM) running R.

**Fig. 2:**
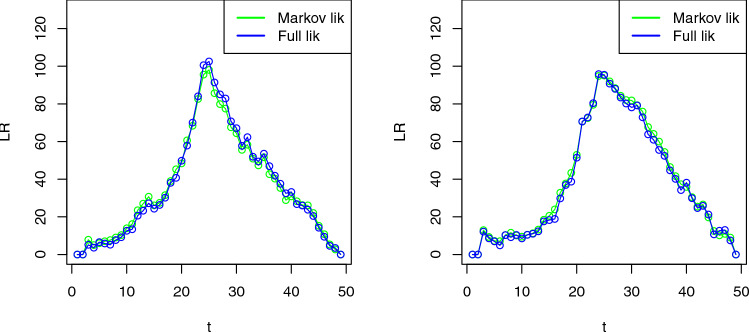
The comparison of first-order Markov likelihood with full likelihood ratio test statistics for detecting a single changepoint in spatial range for the proposed method for two simulated examples

**Table 2:** Evaluation of changepoint detection methods for a single changepoint in the spatial mean. The table compares the performance, measured in terms of Power and True Positive Rate (TPR) across different sizes of change in the spatial mean parameter. Standard errors from 100 simulations are shown in brackets

size of change	Method	Power	TPR
0.25	Proposed (Markov Lik)	0.40 (0.05)	0.25 (0.04)
	Proposed (Full Lik)	0.45 (0.05)	0.30 (0.05)
	Ind Segment (Pair Lik)	0.30 (0.05)	0.05 (0.02)
0.5	Proposed (Markov Lik)	0.84 (0.04)	0.81 (0.04)
	Proposed (Full Lik)	0.86 (0.03)	0.85 (0.04)
	Ind Segment (Pair Lik)	0.69 (0.05)	0.55 (0.05)
1	Proposed (Markov Lik)	1.00 (0.00)	1.00 (0.00)
	Proposed (Full Lik)	1.00 (0.00)	1.00 (0.00)
	Ind Segment (Pair Lik)	0.98 (0.01)	0.95 (0.02)

We further plot the likelihood ratio test statistics for two simulated examples to show the comparison of the Markov likelihood with the full likelihood approach graphically in Figure 2. It can be seen that the test statistic for the Markov likelihood looks similar to the full likelihood approach. We also simulate a Gaussian spatio-temporal process with change in spatial mean with a fixed spatial range, keeping the rest of the simulation settings same. The sizes of the mean change are set at 0.25, 0.5, and 1. The results are presented in Table 2. We see similar results where the proposed method with Markov likelihood outperforms the independent segment method and performs close to the full likelihood approach.

**Fig. 3:**
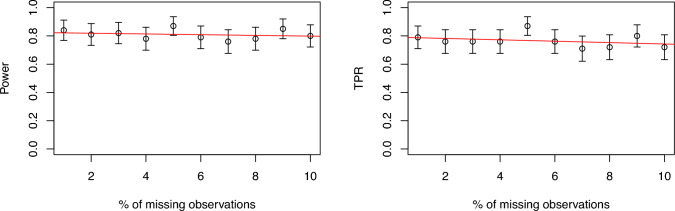
Power and TPR of detecting a single changepoint in mean with Markov assumption as a function of percentage of missing observations in the data. The vertical lines are the 95% confidence bands and the red line is the linear regression fit

In real spatio-temporal data applications it is common to have missing observations. In our simulations, we remove between 1% and 10% of space-time observations completely at random (MCAR). Our method then uses standard conditional Gaussian formulas, subsetting the relevant rows and columns when computing the likelihood. This ensures that the available data still inform the inference process. We examine the impact of missingness on the Markov assumption in a change-in-mean scenario by plotting power and TPR against the proportion of missing observations in Figure 3, along with a fitted linear trend. The fitted linear model shows that the slope of the linear trend is not significant. This provides evidence that within this range of missing data, there is almost no effect on the performance of the Markov assumption in detecting the changepoint.

**Table 3:** Results for a single changepoint in spatial mean with misspecified model comparing different methods in terms of Power and TPR, and evaluated at various sizes of change. Standard errors from 100 simulations are shown in brackets

size of change	Method	Power	TPR
0.25	Proposed (Markov Lik)	0.31 (0.05)	0.16 (0.04)
	Proposed (Full Lik)	0.33 (0.05)	0.19 (0.04)
	Ind Segment (Pair Lik)	0.22 (0.04)	0.04 (0.02)
0.5	Proposed (Markov Lik)	0.69 (0.05)	0.68 (0.05)
	Proposed (Full Lik)	0.71 (0.05)	0.69 (0.05)
	Ind Segment (Pair Lik)	0.58 (0.05)	0.42 (0.05)
1	Proposed (Markov Lik)	0.95 (0.02)	1.00 (0.00)
	Proposed (Full Lik)	0.96 (0.02)	1.00 (0.00)
	Ind Segment (Pair Lik)	0.90 (0.03)	0.93 (0.03)

To demonstrate the robustness of the proposed method under model misspecification, we simulate from the following autoregressive spatio-temporal process (Cressie and Wikle 2015):

$$\begin{aligned} \varvec{Y}_t - \varvec{\mu }_t = \varvec{M}( \varvec{Y}_{t-1} - \varvec{\mu }_{t-1}) + \varvec{\epsilon }_t. \end{aligned}$$

Here $\varvec{Y}_t = (Y_t(\varvec{s}_1), \dots ,Y_t(\varvec{s}_m) )^T$ is the vector representation of the process at *m* spatial locations at time *t* with mean vector $\varvec{\mu }_t$, $\varvec{M}$ is the transition matrix whose $(i, j)^\text {th}$ entry represents how the process at location *j* at the previous time influences the process at location *i* at the current time, and $\varvec{\epsilon }_t$ is Gaussian with mean zero and covariance matrix $\varvec{\Sigma }$. The spatial process is defined on an irregular two-dimensional grid ${\mathcal {S}}$. We choose $\varvec{M}= \text {diag}(\varvec{m})$, where $\varvec{m} = (m(\varvec{s}_1), \dots ,m(\varvec{s}_m) )^T$ allows the autoregressive parameters to vary heterogeneously over space.

We do not change the covariance in time; thus $\Sigma$ remains constant, while the mean $\mu _{t}$ changes abruptly at $t=25$. Specifically, $\mu _{t}(s) = 0$ for $t \le 25$ and $\mu _{t}(s) = \delta$ for $t > 25$. We set $\delta \in \{0.25,\,0.5,\,1\}$ to explore small and larger mean shifts. This isolates the effect of a mean shift even when the true data have autoregressive dynamics. We set the elements of *M* to each be a random value between 0.3 and 0.8 to have heterogeneous simulated data over space. Finally, we simulate a change in mean at $t=25$ and the rest of the simulation settings are the same. The results are presented in Table 3. The proposed method performs reasonably well even in the misspecified model and still outperforms the independent segment method.

Finally, we evaluate the performance of methods for detecting multiple changepoints. We simulate 3 changepoints in mean from the first model with $m= 25$ and $T= 100$. The spatial mean for the four segments is set as (0,-0.5, 0.5, 2). We repeat the simulations 100 times. We report the true positive rate (TPR), the proportion of the correctly identified changepoints out of the true changepoints, false positive rate (FPR), the proportion of incorrect estimators out of the estimated changepoints, and the adjusted rand index (ARI), which measures the similarity between the estimated and true segmentation. The results are reported in Table 4. Note, in particular, how the proposed method outperforms the independent segment method in all the measures.

**Table 4:** Assessment of methods for detecting multiple changepoints in spatial mean using True Positive Rate (TPR), False Positive Rate (FPR), and Adjusted Rand Index (ARI)

Method	TPR	FPR	ARI
Proposed (Markov lik)	0.9167	0.0291	0.9265
Independent Segment (Pair lik)	0.5245	0.0828	0.5129

## Application to Ireland wind data

Wind energy, one of the most economical and sustainable source of energy, is increasingly vital in the global commitment to a more sustainable environment (Wiser et al. 2015). The Irish Meteorological Service (Met Éireann) provides publicly available wind data, crucial for evaluating Ireland’s wind power resource. This data has historical significance and practical utility in wind energy research, as evidenced by previous studies like those of Haslett and Raftery (1989) and Gneiting (2002). Although the Haslett and Raftery (1989)’s study focused on capturing the long memory properties of wind data using fractional differencing, our approach is designed to detect abrupt changes in the spatial process over time rather than explicitly modeling long memory. The Markov approximation employed in our likelihood-based method efficiently focuses on capturing the most impactful short-term dependencies, while still accounting for the overall temporal dynamics.

**Fig. 4:**
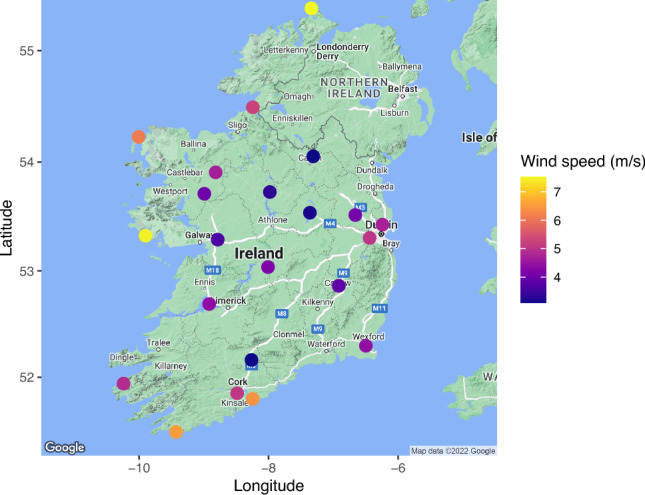
Irish Meteorological Service synoptic weather stations, with mean wind speeds for 1st Jan 2020 to 31st December 2021

**Fig. 5:**
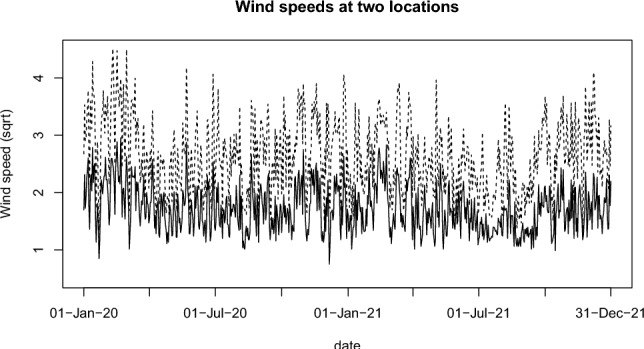
Time series plots for the square root of daily wind speeds at Ballyhaise (solid line) and Malin Head (broken line) for two year period

In this study, we extend this line of research by analyzing daily wind speeds at 22 synoptic weather stations across Ireland from 1st January 2020 to 31st December 2021 (Figure 4). We consider a square root transformation of wind speeds to stabilize the variance and make the marginal distributions approximately normal. Figure 5 shows time series plots of the square root of daily wind speeds at Ballyhaise and Malin Head, stations with the lowest and highest average wind speeds, respectively. Spatial and temporal dependencies are evident in the data. Our focus is to detect and model changes in wind speed patterns, which is not only a critical factor in understanding climate dynamics but also has significant economic implications for wind energy investments (Kalkuhl and Wenz 2020).

The Irish Meteorological service records wind speed data across the country which is vital to evaluate country’s wind power resource. Before starting to install a new wind turbine or wind farm at a potential site, it is usual to record wind data for a short period of time. An estimator based solely on such short-run data at a single station is often inadequate, see e.g. Haslett and Raftery (1989). For such an objective spatio-temporal modelling plays an important role, e.g., using data from synoptic weather stations across the country over a time period to provide an estimate of wind speed at the new site. Moreover, wind speed tends to have strong spatial correlations and exhibit changes of sizeable magnitude over time (Ezzat et al. 2018, 2019). Therefore, it is important to detect these changes and apply a spatio-temporal model which allows for these changes. Here, our objective is to detect changepoints in the wind speed patterns over Ireland that may have useful environmental and economical consequences, and fit a nonstationary model for spatio-temporal prediction. We now continue the analysis of daily Ireland wind speed data at 22 synoptic weather stations over a two year period.

**Fig. 6:**
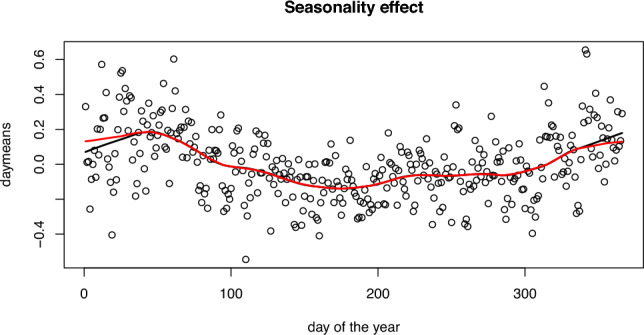
Seasonality effect estimated using 5 years of historical data from 2015-2019. The red line depicts the seasonality effect with smoothing at the edges, while the black line shows the effect without smoothing

To ensure that the changes we may detect are not attributed to seasonal changes, we first estimate the seasonal effect of wind speed and remove seasonality from the data. To estimate the seasonal effect, we use 5 years of historical daily wind speed data, from 1st January 2015 to 31st December 2019. For each day of the year, we compute the average of the square roots of daily wind speeds across all locations and years. After adjusting these averages by subtracting the overall mean, we further explore seasonal trends. This is achieved by plotting the adjusted daily averages against the day of the year, followed by applying the LOWESS (Locally Weighted Scatterplot Smoothing) technique, enabling a detailed observation of the patterns across the annual cycle. To ensure a smooth estimate of seasonality at the edges, we consider data from December (January) also to regress the results in January (December). The estimated seasonal effect is plotted in Figure 6. We subtract the estimated seasonal effect from the data, resulting in seasonally adjusted wind speed measurements. These adjusted measurements are henceforth referred to as adjusted wind speed data.

Focusing on the deseasonalized data, we look at autocorrelation plots of the adjusted wind speed data to get an idea of the temporal dependence. The autocorrelation functions for six weather stations are shown in Figure 9. The adjusted wind speed data exhibit short memory temporal correlations which decay rapidly in time, and they are negligible beyond the time lag of 3 days for most stations. So a third-order Markov assumption is reasonable for the considered dataset. While some stations exhibited longer autocorrelations, the third-order Markov assumption was primarily adopted due to its balance between capturing significant temporal dependencies and maintaining computational feasibility. We also compute empirical spatial correlations of adjusted wind speed data at time lag 0 as an exploratory analysis. For 22 meteorological stations, we plot the empirical spatial correlations for all 231 pairs of stations as a function of spatial distance in kilometers in Figure 10. The adjusted wind speed data at different stations are highly correlated and the correlation decreases as the spatial distances increase. We fit an exponential covariance function: $\sigma ^2 \text {exp}(-c \Vert h\Vert )$ to the empirical correlations using a general least squares estimator and get $\hat{\sigma }^2 = 0.9606$ and $\hat{c} = 0.00113$. The estimated exponential covariance function fits well to the empirical correlation and is also plotted in Figure 10.

In our application, we are concerned with detecting changes in the wind speed over a region and not just a particular station. Such an approach can be relevant as shifts in wind speed patterns taking place over a region can be important to study climate change. The number of stations is $m=22$, and observed daily for 2019-2020 so the total number of time points is $T=731$. There are 5 missing observations in the data, so there are total $n= 16077$ observations. We apply the proposed spatio-temporal changepoint model defined in Section 2.2 to the adjusted wind speed data with exponential spatial covariance. The proposed method allows for missing data by constructing the likelihood with the available observations and their covariances.

The results for detecting changepoint in the spatial process of Ireland wind data are as follows. The proposed method combined with binary segmentation concludes that there exists one changepoint on $24^\text {th}$ July 2021. The threshold was computed using the Monte Carlo method using 100 samples as described in Section 2.2.

**Fig. 7:**
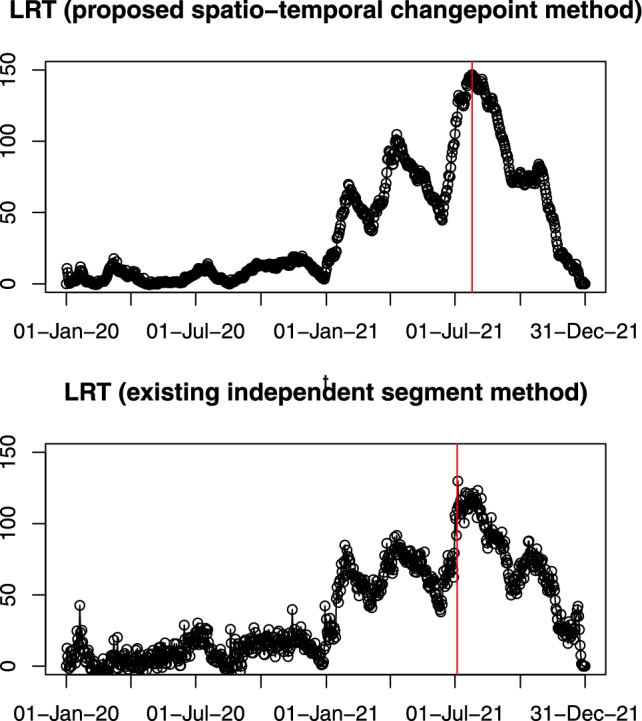
Likelihood ratio test statistics for changepoint detection in adjusted wind speed data over two years. The x-axis represents time with marked date labels, and the y-axis shows the corresponding Likelihood Ratio (LR) values. The upper panel demonstrates the proposed method, while the lower panel shows results from the existing independent segment method. Red lines mark detected changepoints, emphasizing significant shifts

We compare our proposed method with the existing independent segment method to assess their performances in detecting changepoints in the spatial process of adjusted wind speed data. We plot the likelihood ratio statistics for both methods as functions of each candidate time point. These are shown in Figure 7, where the detected changepoints are marked in red for each method. The way the likelihood ratio test statistics vary with changepoint location is qualitatively similar for both methods. The main difference is that the method that assumes independence across segments is, locally, more variable. The explanation for this is that part of the signal for a change at time *t* is based on a shift in the spatial model before and after *t*, and part is from the evidence for independence between data at time *t* and time $t+1$. The first of these is common to both methods, and is the reason for the similarities, but the latter only contributes to the independent segment method. It is this latter contribution, which causes the extra variation. For applications like this, this variation is likely to not be related to the type of change we are wishing to detect, as we do not believe a change would introduce independence between the segments. Interestingly, in this case, the effect of this additional noise is to change the estimated location of the changepoint to 4th July 2021.

For our method, we also tried out the optimistic search method (Kovács et al. 2020) to speed up the detection of the changepoint location, as a full grid search requires fitting the proposed spatio-temporal changepoint model at each candidate changepoint, i.e., 730 times. Optimistic search finds the same changepoint as with the full search and takes only 16 evaluations, making the search roughly 45 times faster. This optimistic search strategy can be very useful for other applications of the proposed method with large time series as it scales with the logarithm of the number of time points.

Once we have detected a changepoint, we can look at the parameters of the spatial process before and after the changepoint to understand the type of change. For the change detected with our method, this suggests that the spatial mean of the adjusted wind speed measure decreases and the spatial range increases after the changepoint. Wind speed is caused by air moving from high pressure to low pressure usually due to changes in temperature. Our detected changepoint coincides with the climate statement of July 2021 on heatwaves from the national meteorological service of Ireland (Met Éireann - The Irish Meteorological Service 2021). Met Éireann state that between the $17^\text {th}$ and $25^\text {th}$ July, the high-pressure system intensified over Ireland blocking any Atlantic weather fronts from approaching, which lead to heatwaves in many places. The changepoint on 24th July 2021 aligns with significant atmospheric changes reported by Met Éireann, indicating a shift in wind dynamics during a period of intense heatwaves. This suggests that such changepoints could serve as indicators of larger climatic shifts, providing valuable insights for climate modeling and environmental forecasting.

While the official heatwave alert did have an end date, no other changepoint emerged as statistically significant within the likelihood ratio test framework. One potential explanation is that after the high-pressure system began, the return to lower temperatures (and higher wind conditions) was more gradual, thus failing to produce an abrupt shift in the spatio-temporal mean or covariance. In fact, Met Éireann’s August 2021 climate statement noted that after a dry start, pressure gradually dropped, highlighting that conditions reverted more smoothly rather than abruptly. As a result, the LR statistic did not reach the threshold required to flag a separate changepoint.

**Fig. 8:**
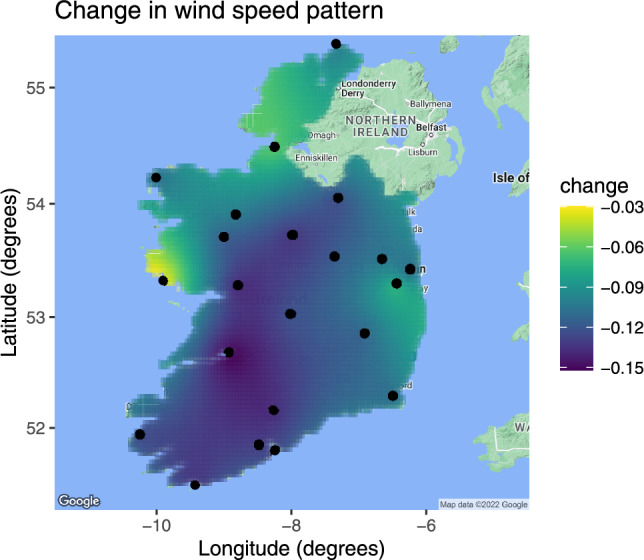
Spatial map showing the change in average square root wind speeds across Ireland before and after the changepoint on 24th July 2021. Each dot represents an Irish Meteorological Service weather station, and the map is generated using ordinary kriging with exponential correlation to visualize the spatial distribution of wind speed changes over Ireland

**Fig. 9:**
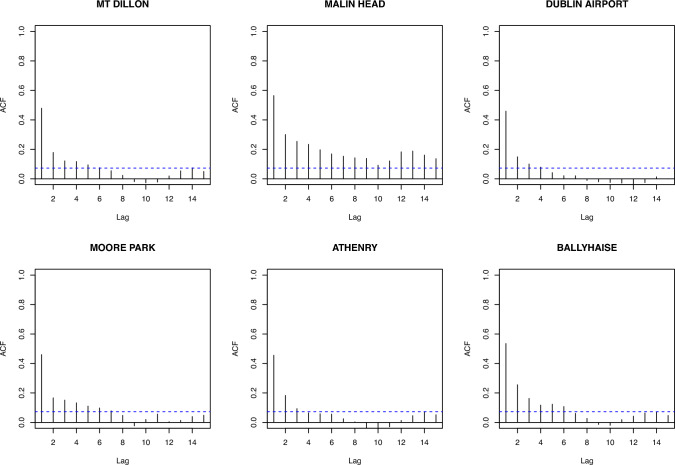
Autocorrelation plots of adjusted wind speed data for 6 synoptic weather stations

**Fig. 10:**
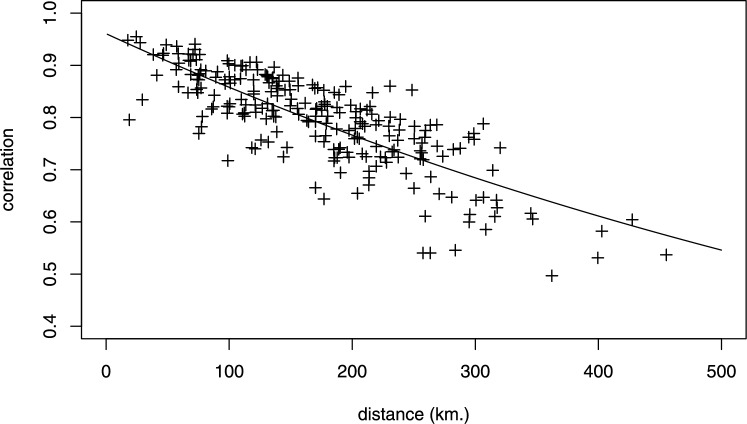
Empirical spatial correlations of each pair of synoptic stations at time lag 0 as a function of spatial distance in kilometers. The solid line is the fitted exponential covariance function

The pattern of the change in wind speed is shown in Figure 8. To visualize the change in the spatial distribution of wind speed, we obtain a spatial map over Ireland using ordinary kriging with the exponential covariance parameters estimated in our spatio-temporal changepoint model during the LRT process. The biggest changes are in the area around Shannon airport and Athenry, while areas around Mace head and Dublin do not show large changes.

Finally, as a way to quantitatively compare our estimated changepoint location with that of the independent segment method, we looked at the accuracy of predictions of held-out data. To do this we fitted a nonstationary spatio-temporal changepoint model to predict wind speed at unobserved locations and time points. The optimal prediction is given by a kriging predictor (Cressie and Wikle 2015), which is the weighted combination of the observed data. The weights are defined by the covariance process, and we compare the fitted covariance matrices from a model with no change, our model with a change on 24th July 2021, and the independent segment model with a change on 4th July 2021. To evaluate the prediction performance, we employ a cross-validation strategy. The testing data consists of random points in both space and time, specifically chosen from the interval between the two changepoints detected by the independent segment method and our proposed method. We focussed on this period in time as it would more clearly highlight any difference between the two estimated changepoints. The remaining data is used for training. Kriging gives us the prediction value $\hat{Y}(\varvec{s}_0, t_0)$ and prediction variance $\hat{\sigma }^2_{\varvec{s}_0, t_0}$ at unobserved $Y(\varvec{s}_0, t_0)$. Under a Gaussian assumption, the predictive distribution is a conditional Gaussian distribution given by $N(\hat{Y}(\varvec{s}_0, t_0), \hat{\sigma }^2_{\varvec{s}_0, t_0})$. To quantify the prediction performance, we use the following commonly used metrics: the root mean square error (RMSE), the continuous ranked probability score (CRPS) (Gneiting et al. 2007), logarithmic score (logS) (Gneiting and Raftery 2007). While RMSE evaluates the accuracy of predicted means, CRPS and logS evaluate the performance of the entire predictive distributions. Lower values of RMSE, CRPS and logS indicate better predictions. The results for the prediction performance of the no changepoint method, the existing changepoint method, and our proposed changepoint method are reported in Table 5. The joint assessment of the metrics shows that the proposed changepoint model has better overall prediction performance as indicated by the lower values of RMSE, CRPS, and logS. While the differences in prediction metrics between models may seem modest, they are statistically meaningful and underscore the enhanced predictive accuracy of our proposed changepoint detection model. This is especially true in domains where small improvements can lead to better risk management and resource allocation. For instance, a lower CRPS and logS indicate not only more accurate predictions but also predictions with better probabilistic calibration, which are crucial for making informed decisions in wind energy management.

**Table 5:** Prediction results for Ireland wind data using no changepoint model, existing independent segment changepoint method, and our proposed changepoint method based on metrics RMSE, CRPS and logS

Metric/Method	No changepoint	Existing method	Proposed method
RMSE	0.54	0.55	0.52
CRPS	0.32	0.32	0.29
logS	0.87	0.83	0.83

## Discussion

The present study proposed a likelihood-based method for detecting changepoints and model parameters within spatio-temporal processes. This new method can potentially be used to study climate change and environmental applications. The proposed method was compared with existing methodologies through a set of simulation studies, and its effectiveness was demonstrated through an application to the daily wind speed measurements collected across different synoptic weather stations in Ireland over a two-year period.

The results of the simulation studies showed that the proposed method performed well in detecting changepoints and estimating model parameters, especially in comparison to existing spatial changepoint methods that assume independence across segments. This finding is particularly important because it suggests that the proposed method can provide more accurate and reliable results for spatio-temporal data, which can have complex structures and dependencies. The simulation results also showed that the computationally-efficient Markov approximation is reasonably accurate and the method is robust in handling datasets with missing observations.

While the Markov likelihood approach offers robust advantages, particularly in handling missing data for Gaussian processes, it is not without limitations. This approach may struggle with accuracy when significant data is missing at recent time steps, possibly requiring the use of data from earlier times which may not accurately reflect current conditions. Furthermore, the method encounters difficulties with non-linear dynamics and long-range dependencies in the data, where the presence of noise can significantly complicate model specification. This necessitates a careful choice in the number of lags included in the model to accurately capture the underlying processes.

We applied our method to daily wind speed data collected from 22 synoptic weather stations in Ireland over a two-year period. The results indicate the presence of one changepoint on 24th July 2021. This information could be useful for studying climate change and wind energy applications, such as wind power resource evaluation. By detecting changes in wind speed patterns over a region, we can identify shifts in wind speed patterns that may have significant environmental and economic consequences. For example, a decrease in wind speed can affect the distribution of pollutants and particulate matter in the atmosphere, which can have negative impacts on human health and the environment.

Future research could focus on adapting our methods to better capture long-term dependencies, which are prevalent in environmental data where the effects of events may unfold over extended periods. Additionally, applying our methodology to other domains such as urban planning and healthcare could yield valuable insights into the spatial dynamics of urban growth and disease spread, respectively. These fields stand to benefit significantly from advancements in spatio-temporal analysis, potentially broadening the impact of our work beyond traditional environmental applications.

## Supplementary information

The datasets and codes that support the findings of this study are openly available on https://github.com/gaurav-agarwal-github/spatio-temporal-changepoint.

Our implementation relies on R (R Core Team 2021) and the following additional R packages: doParallel (Corporation and Weston 2020), fields (Nychka et al. 2021), ggmap (Kahle and Wickham 2013), maps (Richard A. Becker et al. 2021), mgcv (Wood 2011), mvtnorm (Genz et al. 2020), rgdal (Bivand et al. 2022), rlist (Ren 2021), scoringRules (Jordan et al. 2019), and viridis (Garnier 2018).

## Data Availability

The datasets and codes that support the findings of this study are openly available on https://github.com/gaurav-agarwal-github/spatio-temporal-changepoint.
